# Association between area deprivation and major depressive disorder in British men and women: a cohort study

**DOI:** 10.1136/bmjopen-2018-027530

**Published:** 2019-11-25

**Authors:** Olivia Remes, Louise Lafortune, Nick Wainwright, Paul Surtees, Kay-Tee Khaw, Carol Brayne

**Affiliations:** Department of Public Health and Primary Care, University of Cambridge, Cambridge, UK

**Keywords:** mental health, public health, epidemiology

## Abstract

**Objective:**

Studies have shown area-level deprivation can increase the risk for mental disorders over and above individual-level circumstances, such as education and social class. The objective of this study is to determine whether area deprivation is associated with major depressive disorder (MDD) in British women and men separately while adjusting for individual-level factors.

**Design:**

Large, population study.

**Setting:**

UK population-based cohort.

**Participants:**

30 445 people from the general population aged 40 years and older and living in England consented to participate at study baseline, and of these, over 20 000 participants completed a structured Health and Life Experiences Questionnaire used to capture MDD. Area deprivation was measured in 1991 using Census data, and current MDD was assessed in 1996–2000. 8236 men and 10 335 women had complete data on all covariates.

**Primary outcome measure:**

MDD identified according to the Diagnostic and Statistical Manual of Mental Disorders, fourth edition (DSM-IV).

**Results:**

In this study, 3.3% (339/10 335) of women and 2.1% (177/8236) of men had MDD. Men living in the most deprived areas were 51% more likely to have depression than those living in areas that were not deprived (OR=1.51, 95% CI 1.01 to 2.24; p=0.043), but the association between deprivation and MDD was not statistically significant in women (OR=1.24, 95% CI 0.93 to 1.65; p=0.143).

**Conclusion:**

This study shows that the residential environment differentially affects men and women, and this needs to be taken into account by mental health policy-makers. Knowing that men living in deprived conditions are at high risk for having depression helps inform targeted prevention and intervention programmes.

**Strengths and limitations of this study**

- We used a population-based sample of over 20 000 British adults and controlled for important confounders, including social class, medical history and disability.
- We used a structured questionnaire (the Health and Life Experiences Questionnaire of the European Prospective Investigation of Cancer-Norfolk [EPIC-Norfolk] study) to determine whether participants met criteria for major depressive disorder according to the Diagnostic and Statistical Manual of Mental Disorders.
- We used the Townsend index to assess area deprivation. This index is commonly used by researchers to examine deprivation and is a theoretically sound measure.
- People who took part in EPIC-Norfolk were generally more affluent and healthier than those living in other parts of England. As such, our results may not be generalisable to the most deprived areas.

## Introduction

Depression is a common psychiatric disorder affecting approximately 350 million people around the world.^1^ According to the Global Burden of Disease Study,^2^ major depressive disorder (MDD) is a major contributor to the burden of ill health, and other research has shown that it can increase the risk for impairment, disability and suicide.^3–5^ It has also been linked to decreased work productivity, poor quality of life and high health service use.^3 6 7^

A number of studies have examined the individual-level risk factors of depression, such as, family history of mood disorders,^8^ genetics,^9^ history of trauma and stressful life events,^10 11^ and socioeconomic status.^12^ However, the environment or living context can substantially impact our health and influence us over and above individual-level factors.^13–15^ In a systematic review^16^ of 14 studies, 7 found a link between neighbourhood socioeconomic conditions and depression. Living in an area of low socioeconomic status can expose people to a high number of stressors, such as, unsafe neighbourhoods and noise pollution, and this can have deleterious effects on mental health (and self-rated overall health).^17^

There is a wealth of literature on the effect of the places where people live on mental health. Findings from systematic reviews^18–20^ assessing neighbourhood characteristics and depression show that there is large heterogeneity in findings, because of differences in study populations, the confounders that are adjusted for in analyses, and the measures and definitions used to delineate neighbourhoods.^19^ Although there is much evidence on the influence of area-level disadvantage or deprivation on depression, research on this relationship from a gendered perspective is lacking.

We used data from a large, population-based, cohort study to determine the influence of area deprivation on risk of having MDD in men and women separately, while adjusting for a range of important confounders, including social class, medical conditions, psychiatric comorbidity and disability. In this research, area deprivation is defined by living contexts in which there are high levels of: unemployment, non-home ownership, non-car ownership and low income.^13^ Findings are disaggregated by gender, and this is done for several reasons. Gender can serve as a gateway to resources derived from the environment.^21 22^Compared with men, women have been shown to have fewer financial resources, lower social status, and less power, and this can have a detrimental impact on mental wellbeing. Women have typically suffered from discrimination, and because of this, have been exposed to fewer opportunities for education, training, and adequate employment.^23^ Women have also taken on different job roles and tasks than men, which has exposed them to different hazards and contaminants affecting their health. Furthermore, they have traditionally been seen as ‘care-takers’ in society and involved in domestic work, which might have interfered with their education or career paths. As such, they might have had less access to the necessary resources with which to achieve good health and wellbeing.^23 24^

However, there are additional reasons why findings are disaggregated by gender. Women and men tend to be affected by different kinds of stressors. Recent research has shown that men are more susceptible to stressors related to work and finances as well as legal issues, while women are more affected by the relationships they have (or lack thereof) and interpersonal difficulties.^25 26^ These are some of the findings reported by a study conducted in the USA and a review of results from various parts of the world. Hence, living in a deprived area with high levels of unemployment might be particularly detrimental for men’s mental health. This was evident when the economy shifted in the UK from a manufacturing-- to a service-based one, and men lost their jobs.^27^ Prior to the shift, men typically performed skilled and semi-skilled jobs, which significantly contributed to the local economy. After the economic shift, a larger number of women entered employment (occupying mainly service industry jobs); this change likely had a significant impact on gender social roles. Men who experienced fewer financial and job opportunities may have suffered from lowered self-worth and their self-concept may have been negatively affected; loss of social position and role identify may have had consequences for their physical and mental health.^27^ A recent study^25^ showed that men’s mental health is particularly affected if they fail at key instrumental tasks, such as, inability to achieve (ex. to reach goals, such as those related to work and finances, which can sustain family life). In contrast, women are more likely to be depressed if they fail to meet their needs for relationship.^25^ To this end, it appears that men and women are susceptible to different kinds of stressors.

It remains unclear whether men and women living in areas of above average deprivation are differentially susceptible to MDD—the objective of this study will be to assess this. Determining whether one gender is at risk of having depression when exposed to deprived circumstances helps to inform interventions and target scarce health resources to population sub-groups.^28^

## Methods

Data were drawn from EPIC-Norfolk, whose design and study methods have been described in detail elsewhere.^29^ In brief, a prospective population-based cohort of 30 445 participants ages 40–74 years were recruited by post between 1993 and 1997 through general practice age-sex registers in the city of Norwich and the surrounding small towns and rural areas. At baseline (1993–1997), participants completed a postal Health and Life Experiences (HLQ) questionnaire that captured information on sociodemographics, including age, gender, highest educational attainment, marital status, social class, employment status, ethnicity and self-reported physician diagnoses of physical diseases. Using participants’ postal codes, a measure of area deprivation was derived based on the 1991 Census. Between 1993 and 2000, participants completed self-reported postal questionnaires provided they: (1) were still alive, (2) did not ask to be removed from the study’s mailing list and (3) had a valid mailing address.

During 1996–2000, 20 919 participants completed a structured, psychosocial Health and Life Experiences Questionnaire (HLEQ). During this time, an assessment of generalised anxiety disorder (GAD) and MDD was made according to the Diagnostic and Statistical Manual of Mental Disorders, fourth edition (DSM-IV).^30^ Using the HLEQ, disability measures based on the SF-36 were also derived.^31^

All participants recruited through general-practice registers and who completed a baseline health questionnaire were eligible to be included in our study; those who completed a psychosocial questionnaire during follow-up were eligible to be included in our analysis.

In regards to the study size, an initial sample of 30 445 participants completed the baseline HLQ and of these, 20 921 filled out the psychosocial HLEQ. After retaining the people with complete measures on all covariates, the final sample size was 18 571.

Although EPIC-Norfolk is a prospective study and area derivation was measured in 1991 and anxiety in 1996–2000, this analysis should be considered cross-sectional.

### Dependent variable

The primary outcome in this study was current MDD, which was measured using the HLEQ. The HLEQ is a structured psychosocialquestionnaire part of a large epidemiology project; it is designed to capture self-reported data, such as that on depression.^32 33^ DSM-IV criteria were applied to the psychiatric symptoms captured using the HLEQ in order to determine whether participants had an episode of MDD that was ongoing at the time of the completion of the questionnaire. Participants who reported a psychiatric episode were asked to estimate the onset and offset timings of the episode, and then to report an outline of the history of the problem. Participants were also asked about age at first symptom onset and subsequent episode recurrence.

The dependent variable in this study is current MDD, defined as an episode of MDD reported as ongoing at the time of the completion of the HLEQ.

The following two core criteria of MDD were first evaluated:

1. Have there ever been times in your life when you felt sad or depressed for 2 weeks or more in a row?
2. Have there ever been times in your life when you lost interest in most things like your work or activities that usually give you pleasure, for 2 weeks or more in a row?

If participants answered yes to one of these questions, they were then asked to think of the most recent 2-week episode during their lives when these feelings of sadness, depression or loss of interest were the worst. They then had to report that these feelings of being sad, depressed, or loss of interest lasted all day or most of the day, and that during these 2 weeks of their most recent episode, they felt this way every day or almost every day.

In addition, at least five of the following symptoms had to be present: gaining or losing weight, having trouble falling asleep or sleeping too much, feeling tired or low on energy, feeling unable to sit still or feeling slowed down, experiencing guilt or shame, feeling worthless, losing confidence, having trouble concentrating and thinking a lot about death or suicide. One of these five symptoms had to be one of the two core criteria evaluated at the beginning.

Finally, it was evaluated whether these symptoms interfered with participants’ lives and resulted in help-seeking or impairment.

### Individual-level measures (potential confounders)

Individual-level measures included age, education, employment status, marital status, social class, health status, ethnicity, and history of anxietyand disability. The final categorisation of the variables took cell size into account and was also done in accordance with previous literature.^33–39^ Age was divided into 10-year bands. Educational attainment was categorised into high (vocational or formal qualifications at the A-level or O-level or degree-level qualifications) versus low (no formal qualifications). Further details on the meaning of A-level and O-level can be found elsewhere^40 41^; the appendix also contains definitions of these (online supplementary appendix 1). Employment was divided into yes versus no. Marital status was categorised into three groups: married, single (or never married) and others (widowed, divorced, separated). Social class was derived using the Computer-Assisted Standard Occupational Coding^42^ and categorised as follows: I (professionals), II (managerial and technical occupations), III non-manual and III manual (skilled workers), IV (partly skilled workers) and V (unskilled manual workers). To assign social class to men and women, the male partner’s current or past occupation was used. If this information was not available, the female partner’s occupation was used. If the social class from either partner was unavailable, then it was coded as missing. The final categorisation of social class included manual: skilled manual, partly skilled and unskilled; and non-manual: professionals, managerial and technical, and skilled non-manual. Individual-level health status was assessed through the construction of a variable capturing major prevalent physical diseases. This was based on HLQ questions asking participants: "Has the doctor ever told you that you have any of the following?”, followed by a list of options, such as allergies, asthma, cancer, stroke, heart attack, diabetes, thyroid conditions, and so on. Ethnicity was based on a self-reported question asking participants to tick the relevant box: ‘white’, ‘black Caribbean’, ‘black other’, ‘Indian’, ‘Pakistani’, ‘Bangladeshi’, ‘Chinese’, ‘other’.

10.1136/bmjopen-2018-027530.supp1 Supplementary file 1

Lifetime history of GAD was also assessed using the self-reported HLEQ questionnaire.^33^ Lifetime GAD consisted of having ever had at least one episode that met core criteria stipulated by the DSM-IV. Anxiety in EPIC-Norfolk was identified if participants reported having uncontrollable, excessive worry for 6 months or longer on most days than not that resulted in help-seeking or impairment. In addition, at least three of the following symptoms needed to have been present: restlessness, irritability, muscle tension, fatigue, trouble concentrating because of worry, mind going blank, trouble falling asleep, trouble staying asleep and feeling keyed up or on edge.

To determine disability levels, we used the physical component summary (PCS) derived from the HLEQ. The PCS is part of the SF-36, a widely used, validated self-assessment tool. The SF-36 is a 36-item measure capturing eight health dimensions: physical functioning, social functioning, role limitations due to physical problems, role limitations due to emotional problems, mental health, energy/vitality, bodily pain and general health perception. The eight dimensions of the SF-36 were used to create two higher order scores, one of which was the PCS. Higher scores indicate better health.^31^

All of these individual-level variables were viewed as potential confounders and selected based on the research literature and their links to depression and area-level socioeconomic circumstances.

### Area-level measure (exposure variable)

To examine area deprivation, we used the Townsend Index.^43 44^ This is one of the most commonly used measures of area deprivation in the UK and particularly appropriate for the time of the original EPIC-Norfolk study. This index is a composite measure of four variables obtained from the 1991 Census: (1) percentage of economically active residents over age 16 who are unemployed, (2) percentage of households that do not possess a car, (3) percentage of private households that are not owner occupied and (4) percentage of private households that are overcrowded (have more than one person per room). These variables were obtained at the level of the enumeration district, which is a geographic area used for census purposes in Britain. Each variable was standardised by obtaining Z scores (dividing the mean by the SD across enumeration districts in England). The Z values of the four variables were added together to produce a Townsend index score for each enumeration district. A score of 0 represents the national mean, while positive values of the index indicate enumeration districts that are above average deprivation, while negative values indicate those that are below average deprivation. The postal codes of participants were record linked to enumeration districts, and participants were considered to live in areas of above average deprivation depending on the Townsend index score assigned to their enumeration district.^43^

Depending on the results from the main analysis (association between overall area deprivation and depression), the Townsend deprivation index was disaggregated into its four constituent components to determine whether any one of these is associated with MDD.

### Missing data

The number of missing observations for each covariate were: nine for education, 47 for marital status, 417 for MDD, 434 for GAD, 458 for social class, 75 for the Townsend index and 1386 for the SF-36, 52 for employment status.

### Statistical analysis

First, we compared participants on sociodemographic, and medical and psychiatric history characteristics, and the prevalence of MDD was computed for subgroups. Next, we undertook correlated data analysis based on generalised estimating equations (GEE)^45 46^ to determine the population-average effect of living in an area of above average deprivation on risk of having depression while controlling for confounders. MDD is a dichotomous outcome and the intra-cluster correlation was assumed to be equal. As such, we used GEE with a logit link and an exchangeable correlation structure.

First, we ran unadjusted analyses between deprivation and MDD. To determine the influence of potential confounders on risk of having depression, we progressively adjusted the models and accounted for (1) age, educational attainment, marital status, social class, and employment; then for (2) age, educational attainment, marital status, social class, employment, and GAD; and finally for (3) age, educational attainment, marital status, social class, employment, GAD, physical diseases and disability level. We conducted separate analyses for men and women. The individual-level covariates were sociodemographics, and medical and psychiatric history, while the area-level covariate was the Townsend index score. The progressively adjusted models allowed us to estimate adjusted ORs and 95% CIs.

A dichotomous variable was created using the Townsend index scores, and 0 was used as the cut-point (considered to be the national average). The variable was dichotomised, because we wanted to compare participants’ scores to the national average^47^—scores above the cut-point of 0 were considered above average deprivation. A binary variable was also used in accordance with previous research^47^ and because of cell size considerations—we wanted to ensure that there were sufficient people with MDD in each category of the deprivation variable.

Models were constructed for participants with complete measurements on all covariates. It was not feasible to re-categorize the MDD variable, because it was created according to the DSM-IV classification;^32 33^ area deprivation was examined in line with other studies.^37 43^

Several sensitivity analyses were undertaken. We ran fully adjusted models using pure MDD as the outcome, in which those with past-year GAD were excluded. It should be mentioned that although GAD and MDD have been regarded as closely correlated by many researchers, they are independent disorders. The high GAD-MDD comorbidity found in older literature was due to the use of clinical populations with multiple co-occurring conditions.

Next, we disaggregated the index used to measure disadvantage. If a significant relationship was found between area deprivation and depression for one of the genders in a fully adjusted model, we investigated further. We disaggregated the Townsend index into its four constituent components (unemployment, non-home ownership, non-car ownership and overcrowding) to determine whether any aspect of deprivation is associated with increased risk of having depression in that gender group. Each component was dichotomised using a cut-point of 0, because it represents the national average.

Then we determined whether relationships held after dividing the Townsend index into quintiles and adjusting for sociodemographic and health status variables. Further, we examined whether the inclusion of additional covariates or recategorisation of variables made any difference to the effect estimates. We included ethnicity as a potential confounder in a fully adjusted model, and assessed whether the division of the education variable into four categories influenced the associations.

Finally, we conducted logistic regression, which does not take the intracluster correlation into account, and compared the findings to those from GEE. Similar results between the models suggests that the intraclass correlation is negligible.

All models used two-sided statistical tests, and a p value of <0.05 was considered statistically significant. Statistical Analysis Software (SAS) V.9.3 was used in these analyses.

### Patient and public involvement

There were no patients or public involved in the development of the research question, outcome measures, design of the study, or recruitment to and conduct of the study.

## Results

At baseline, 30 445 participants were recruited from general practices in the city of Norwich and the surrounding towns and rural areas. Of these, 20 919 people completed the HLEQ during the follow-up period. In total, 18 571 out of 20 919 (89%) people were available for analysis, because they had complete data on all covariates.

In this sample, there were 8236 men and 10 335 women over the age of 40 years. Table 1 shows the distribution of individual-level and area-level characteristics by current MDD.

**Table 1:** Distribution of characteristics for women and men who completed the Health and Life Experiences Questionnaire in the EPIC-Norfolk cohort

Characteristic	Women (n=10 335)		Men (n=8236)	
	Number with characteristic	Percentage and number with MDD	Number with characteristic	Percentage and number with MDD
**Individual-level variables**				
**Sociodemographics**				
Age (years)				
<50	1450	5.0 (72)*	964	3.4 (33)*
50–60	3716	3.9 (145)	2651	3.0 (80)
60–70	3180	2.1 (68)	2743	1.5 (40)
>70	1989	2.7 (54)	1878	1.3 (24)
Education†				
Low	4050	3.5 (141)	2365	2.2 (51)
High	6285	3.2 (198)	5871	2.1 (126)
Marital status				
Single	417	2.4 (10)*	303	3.6 (11)*
Married	7750	2.7 (207)	7237	1.7 (122)
Other‡	2168	5.6 (122)	696	6.3 (44)
Social class§				
Manual	3829	3.3 (127)	3286	2.3 (76)
Non-manual	6506	3.3 (212)	4950	2.0 (101)
Employment				
Yes	4075	3.1 (128)	3821	1.8 (68)*
No	6260	3.4 (211)	4415	2.5 (109)
**Health status**				
Prevalent physical disease				
Yes¶	5698	3.8 (214)*	3843	2.6 (100)*
No	4637	2.7 (125)	4393	1.8 (77)
Disability level				
High**	5296	3.9 (208)*	4021	3.0 (119)*
Low	5039	2.6 (131)	4215	1.4 (58)
Lifetime GAD				
Yes	448	19.4 (87)*	255	22.4 (57)*
No	9887	2.5 (252)	7981	1.5 (120)
**Area-level variable**				
Townsend index				
Deprivation				
Above average deprivation (>0)	1646	4.6 (76)*	1242	3.6 (45)*
Below average deprivation (≤0)	8689	3.0 (263)	6994	1.9 (132)

*p<0.001; ***p<0.05.

†High education: O-level, A-level, degree; low education: refers to no education.

‡Other: divorced, separated, widowed.

§Manual: skilled manual, semi-skilled, non-skilled; non-manual: professionals, managerial, skilled non-manual.

¶Prevalent physical disease: respiratory disease (asthma and bronchitis), allergies (allergies and hay fever), stroke, heart attack, cancer, diabetes, thyroid conditions, arthritis.

**Below the PCS value of 50.6.

††See online supplementary appendix 2 for the distribution of the Townsend index scores in men and women.

GAD, generalised anxiety disorder; MDD, major depressive disorder; PCS, physical component summary.

The prevalence of (current) MDD was 2.1% (177/8236) for men and 3.3% (339/10335) for women. Women with MDD were younger than 50 years of age, more likely to be divorced/separated/widowed, have prevalent physical disease, high disability, GAD, and live in areas of above average deprivation. Among men, similar patterns emerged (table 1). Men with MDD were also more likely to be unemployed.

After performing correlated data analysis based on GEE, findings showed that the risk of depression in men living in areas of above average deprivation was 95% higher in an unadjusted analysis (OR=1.95, 95% CI 1.39 to 2.76; p=0.0001) (results not shown). After accounting for sociodemographics, the OR attenuated slightly to 1.57 (OR=1.57, 95% CI 1.09 to 2.26; p=0.0152) (table 2).

**Table 2:** ORs for major depressive disorder according to individual- and area-level characteristics for men (n=8236) who completed the Health and Life Experiences Questionnaire in the EPIC-Norfolk cohort

Characteristic*	ORs and 95% CI					
	Model A†	P-value for model A	Model B‡	P-value for model B	Model C§	P-value for model C
**Individual-level variables**						
Sociodemographics						
Age (per 10 years)	0.40 (0.32 to 0.50)	<0.0001	0.50 (0.40 to 0.63)	<0.0001	0.47 (0.38 to 0.60)	<0.0001
Education¶						
Low	1.10 (0.76 to 1.61)	0.6081	1.06 (0.72 to 1.54)	0.7813	1.00 (0.68 to 1.46)	0.9978
High	1.00		1.00		1.00	
Marital status						
Single	1.46 (0.76 to 2.83)	<0.0001	1.39 (0.71 to 2.68)	<0.0001	1.41 (0.72 to 2.76)	<0.0001
Married	1.00		1.00		1.00	
Other**	3.66 (2.53 to 5.28)		3.48 (2.31 to 5.22)		3.58 (2.39 to 5.35)	
Social class††						
Manual	1.02 (0.73 to 1.41)	0.9161	1.14 (0.81 to 1.59)	0.4612	1.06 (0.76 to 1.48)	0.7298
Non-manual	1.00		1.00		1.00	
Employment††						
No	3.69 (2.48 to 5.50)	<0.0001	2.64 (1.74 to 4.03)	<0.0001	2.24 (1.46 to 3.45)	0.0002
Yes			1.00		1.00	
**Health status**						
Lifetime GAD						
Yes			14.33 (9.84 to 20.87)	<0.0001	12.65 (8.68 to 18.44)	<0.0001
No			1.00		1.00	
Prevalent physical disease						
Yes‡‡					1.25 (0.89 to 1.75)	0.1977
No					1.00	
Disability level						
High§§					1.98 (1.39 to 2.82)	0.0002
Low					1.00	
**Area-level variable**						
Townsend index						
Deprivation						
Above average deprivation (>0)	1.57 (1.09 to 2.26)	0.0152	1.56 (1.05 to 2.31)	0.0287	1.51 (1.01 to 2.24)	0.0434
Below average deprivation (≤0)	1.00		1.00		1.00	

*The brackets show the reference categories that were used for each categorical variable when it was entered in the models—deprivation: below average deprivation (ref) versus above average deprivation; education: high (ref) versus low; marital status: married (ref), single, others; social class: non-manual (ref) versus manual; employment: yes (ref) versus no; lifetime GAD: no (ref) versus yes; prevalent physical disease: no (ref) versus yes; disability level: low (ref) versus high. These reference categories were based on the literature.

†Adjusted for age, sociodemographics (education, marital status, social class, employment status).

‡Adjusted for age, sociodemographics, lifetime GAD.

§Adjusted for age, sociodemographics, lifetime GAD, physical diseases and disability.

¶High education: O-level, A-level, degree; low education: refers to no education.

**Other: divorced, separated, widowed.

††Manual: skilled manual, semi-skilled, non-skilled; non-manual: professionals, managerial, skilled non-manual.

‡‡ Prevalent physical disease: respiratory disease (asthma, bronchitis), allergies (allergies, hay fever), stroke, heart attack, cancer, diabetes, thyroid conditions, arthritis.

§§Above the PCS value of 50.6.

GAD, generalised anxiety disorder; PCS, physical component summary.

The OR reduced slightly after controlling for lifetime GAD (OR=1.56, 95% CI 1.05 to 2.31; p=0.029), but remained highly significant. After additionally adjusting for prevalent physical diseases and disability, the effect estimate became somewhat attenuated (OR=1.51, 95% CI 1.01 to 2.24; p=0.043), however, a statistically significant association between area derivation and depression remained (table 2). As the association with area deprivation emerged to be statistically significant for men (table 2), we took this finding further and wanted to determine the specific component of deprivation that was related to men’s risk of having poor mental health (by disaggregating the Townsend index into its constituent components). Results showed that the OR was highest for unemployment (OR=1.77, 95% CI 1.16 to 2.71; p=0.008), followed by non-car ownership (OR=1.20, 95% CI 0.70 to 2.04; p=0.507), and lowest for overcrowding (OR=0.93, 95% CI 0.60 to 1.42; p=0.727) and non-home ownership (OR=0.81, 95% CI 0.49 to 1.34; p=0.422). Of these, only the effect estimate for unemployment was statistically significant (online supplementary appendix 3). Men living in areas characterised by high levels of unemployment were almost 80% more likely to have depression than those living in areas with low levels of unemployment. Next, we wanted to determine whether deprivation is associated with pure MDD, and thus excluded past-year GAD; the association with depression remained statistically significant (OR=1.64, 95% CI 1.06 to 2.52; p=0.025).

In women, while there was a statistically significant association in the unadjusted analysis (OR=1.55, CI 95% 1.19 to 2.01; p=0.0010) as well as in the model adjusting for sociodemographics (OR=1.40, 95% CI 1.07 to 1.84; p=0.013), the association lost its significance in the fully adjusted model (OR=1.24, 95% CI 0.93 to 1.65; p=0.143) (table 3). Thus, we did not carry out further analyses using the Townsend index.

**Table 3:** ORs for major depressive disorder according to individual- and area-level characteristics for women (n=10 335) who completed the Health and Life Experiences Questionnaire in the EPIC-Norfolk cohort

Characteristic*	ORs and 95% CI					
	Model A†	P-value for model A	Model B‡	P-value for model B	Model C§	P-value for model C
**Individual-level variables**						
Sociodemographics						
Age (per 10 years)	0.54 (0.46 to 0.64)	<0.0001	0.62 (0.52 to 0.74)	<0.0001	0.59 (0.50 to 0.71)	<0.0001
Education¶						
Low	1.23 (0.97 to 1.56)	0.0890	1.29 (1.01 to 1.65)	0.0412	1.30 (1.02 to 1.66)	0.0356
High	1.00		1.00		1.00	
Marital status						
Single	0.93 (0.48 to 1.78)	<0.0001	0.91 (0.48 to 1.75)	<0.0001	0.91 (0.47 to 1.75)	<0.0001
Married	1.00		1.00		1.00	
Other**	2.56 (2.00 to 3.27)		2.41 (1.87 to 3.10)		2.36 (1.83 to 3.04)	
Social class††						
Manual	0.95 (0.75 to 1.21)	0.6964	0.99 (0.77 to 1.27)	0.9530	0.97 (0.76 to 1.25)	0.8225
Non-manual	1.00		1.00		1.00	
Employment††						
No	1.87 (1.42 to 2.48)	<0.0001	1.62 (1.21 to 2.15)	0.0010	1.55 (1.17 to 2.06)	0.0026
Yes			1.00		1.00	
**Health status**						
Lifetime GAD						
Yes			7.97 (5.99 to 10.60)	<0.0001	7.37 (5.52 to 9.83)	<0.0001
No			1.00		1.00	
Prevalent physical disease						
Yes‡‡					1.25 (0.98 to 1.59)	0.0682
No					1.00	
Disability level						
High§§					1.41 (1.11 to 1.79)	0.0045
Low					1.00	
**Area-level variable**						
Townsend index						
Deprivation						
Above average deprivation (>0)	1.40 (1.07 to 1.84)	0.0132	1.26 (0.95 to 1.67)	0.1081	1.24 (0.93 to 1.65)	0.1425
Below average deprivation (≤0)	1.00		1.00		1.00	

*The brackets show the reference categories that were used for each categorical variable when it was entered in the models—below average deprivation (ref) versus above average deprivation; education: high (ref) versus low; marital status: married (ref), single, others; social class: non-manual (ref) versus manual; employment: yes (ref) versus no; lifetime GAD: no (ref) versus yes; prevalent physical disease: no (ref) versus yes; disability level: low (ref) versus high. These reference categories were based on the literature.

†Adjusted for age, sociodemographics (education, marital status, social class, employment status).

‡Adjusted for age, sociodemographics, lifetime GAD.

§Adjusted for age, sociodemographics, lifetime GAD, physical diseases and disability.

¶High education: O-level, A-level, degree; low education: refers to no education.

**Other: divorced, separated, widowed.

**††Manual: skilled manual, semi-skilled, non-skilled; non-manual: professionals, managerial, skilled non-manual.

‡‡Prevalent physical disease: respiratory disease (asthma, bronchitis), allergies (allergies, hay fever), stroke, heart attack, cancer, diabetes, thyroid conditions, arthritis.

§§Below the PCS value of 50.6.

GAD, generalised anxiety disorder; PCS, physical component summary.

We also conducted some sensitivity analyses. First, we divided the Townsend index into quintiles. Results showed that men living in the most deprived quintile had a statistically significantly increased risk for depression (OR=1.68, 95% CI1.01 to 2.79; 0.0472), while none of the quintiles for women showed statistically significant findings. Second, we wanted to determine whether there was any change in findings after incorporating ethnicity in the original fully adjusted models. The associations remained the same (men: OR=1.53, 95% CI 1.03 to 2.27 and women: OR=1.25, 95% CI 0.94 to 1.66). Second, we undertook analyses in which the education variable was left in its original form (divided into four categories: no education, O-level, A-level, degree and beyond) in fully adjusted models, and similar findings were again obtained (men: OR=1.51, 95% CI 1.02 to 2.24) and women: OR=1.23, 95% CI 0.92 to 1.63). Third, we reran the fully adjusted models using logistic regression rather than correlated data analysis based on GEE (online supplementary appendix 4), and results remained essentially unchanged (men: OR=1.51, 95% CI 1.03 to 2.21 and women: OR=1.24, 95% CI 0.94 to 1.64). This shows that there indeed is a robust association between overall area deprivation and depression in men, while there is no statistically significant effect in women.

## Discussion

This research is an analysis based on EPIC-Norfolk data, and findings showed that living in an area of above average deprivation was associated with a significantly increased risk of depression in men; the relationship with depression was not statistically significant in women. The association in men persisted after adjusting for important individual-level confounders, such as serious physical health conditions, disability, and history of GAD. When we looked closer to determine the specific component of area deprivation with the greatest influence on men’s mental health, unemployment emerged as important. Men living in areas characterised by high unemployment had a 77% greater chance of having depression than those living in areas with low levels of unemployment.

### Potential mechanisms

An environment in which deprivation is above average according to the Townsend index appears to differentially affect men and women’s mental health after accounting for a number of potential confounders. A number of reasons can explain this. First, men may be more sensitive to certain stressful events occurring in their environment compared with women, especially if the stress is relating to financial and work-related problems.^25^ If men are still seen as head of household or feel the responsibility to sustain their families, then stressors related to occupation and finances can take a toll on their mental health.Second, when living in disadvantaged regions, the possibility of hearing about job loss from others increases and this can heighten stress in those who are still working, which can increase their risk of depression.^48^ This is particularly problematic for men who are perceived by their families as the main provider. In contrast, women’s risk of depression seems to be influenced more by the social networks they are embedded in and problems stemming from those relations; low parental warmth, divorce, low social support, and low marital satisfaction are interpersonal stressors which can impact women’s mental health.^25 26^ Women may be at risk for having depression if their interpersonal needs are not met. 25 Men, on the other hand, who perceive themselves to fail at key instrumental tasks (such as providing for the family and bringing in income) can be at risk for poor mental health.^25 49^

Unemployment, often accompanied by low social ranking, can negatively affect the self concept and may be a source of role loss in men. There were indications that this social phenomenon took place in the UK after the 1970s, when the economic landscape changed from a manufacturing to a service-based one.^27^ The economic shift was accompanied by job loss in men, while women entered the workforce. This change had a harmful impact on many individuals, and men’s loss of employment might have been tied to loss of self-esteem, among other negative sequelae.^27^ Even more than a decade after this shift in economy took place, occupational class remained important for men’s wellbeing; specifically, their self-rated health was much more affected by work-related factors than sociodemographics, such as education. 50 Recent research further shows that problems in the work domain greatly affect men.^25^ Taken together, this supports the notion that men are affected by failure at key instrumental tasks, especially tasks that may be important in sustaining a family.^25^ A similar phenomenon occurred in rural regions of Midwestern United States after the farm crisis occurred in the 1980s.^51^ Rural areas upheld traditional gender roles, with male provider norms and men showing ‘rugged independence’.^51^ After the farm crisis hit, men had greater difficulty in fulfilling their traditional provider role and the changing economic landscape disrupted the traditional gender-based system. This created stress and contributed to depression in those living in rural villages and small towns of the Midwest. During this time, men showed susceptibility to a wider range of stressors (ex. financial stress, among others) compared with women.^51^

Men and women also tend to experience and manifest the effect of stress in different ways. Women living in deprived areas have been shown to be more prone to anxiety,^28^ while men living in disadvantage are more likely to have depression. This could be a result of evolutionary, survival functions. Women have traditionally had the responsibility of childcare and ensuring the successful survival of future generations.^52^ Therefore, living in circumstances of (above average) deprivation can trigger the fight or flight reaction, which can increase stress in finding ways to make ends meet so that they can raise their children. In this context, anxiety might be seen as protective, ensuring the survival of future generations. This is why women also tend to be more concerned about neighbourhood characteristics that can negatively impact their family and the raising of their children.^52 53^ Men have traditionally had the responsibility of being the provider and to do this, need to maintain a certain socioeconomic status; if they are not able to achieve this, they are more likely to become depressed^25^ ^27^ (and depression is a risk factor for suicide). This is a problem in India, where suicide rates are high among male farmers whose crops have failed (or who experienced problems with crops that led to indebtedness).^54 55^ In the UK, men with depression are also more likely than women to commit suicide.^56^ Taken together, these findings suggest that women may actually be more resilient than men when encountering adversity - women anxiously struggle to ensure the survival of their children and future generations, while men succumb to depression and potentially suicide. However, very little research has examined this, and previous studies in the mental health literature have typically described women as vulnerable. Further research on health from a gendered perspective is needed.^28^

When exposed to the stresses and strains of deprivation, men are also more likely to develop substance abuse and this, in turn, can increase the risk for depression. The National Epidemiologic Survey on Alcohol and Related Conditions study^57^ showed that the total number of stressors experienced in life had a stronger association with heavy drinking in men than in women. Experiencing stressors can also lead to unhealthy means of coping, such as smoking and physical inactivity, and this can lead to sequelae.^24 58^ Finally, when men experience mental health issues, they are less likely to seek help than women.^51^

### Strengths, weaknesses and future research

This study shows that there is a statistically significant association between overall area deprivation and depression in men, while this relationship is not apparent in women. There are a number of strengths associated with our research. Our study used a structured questionnaire, the HLEQ, to assess mental health, and a measure of MDD was created using valid and reliable criteria stipulated by the DSM. Also, we were able to adjust for a number of important confounders, such as medical and psychiatric history, and sociodemographic factors, including unemployment measured at the level of the individual. Nonetheless, residual confounding may be present in our research if certain covariates were not adequately adjusted for. With respect to the medical history covariate, it is possible that some participants may have omitted disclosing or had difficulty recalling medical diagnoses and this might have introduced measurement error. Our measure of area deprivation also may not capture features of the environment that may affect mental health; however, all indexes designed to measure environmental effects suffer from this limitation. The Townsend index is theoretically sound and commonly-used in research assessing these types of relationships. One of the limitations of this variable is that it may be a better measure of deprivation in urban settings, particularly as it is capturing aspects that are more commonly found in or are typical of urban contexts (eg, car ownership). Given that it may not be capturing rural deprivation as well as it should, measurement error may be an issue. This is an area of further research.

Because of healthy volunteer bias, it is possible that some of the sickest, most deprived people who would have been eligible to take part in EPIC-Norfolk, did not participate. This means that our results may not generalise to those individuals.

Also, we did not have information on length of living in the area for participants, however, migration in EPIC-Norfolk is minimal and unlikely to have biased the findings. People who took part in this study tended to reside in the same areas their whole lives. This is why Norfolk and the surrounding towns and rural areas were selected for participant recruitment.^59^

Another issue is the fact that EPIC-Norfolk only included people over the age of 40. As critical time periods for the development of depression include young adulthood,^60^ it would be useful if future research examined these relationships with deprivation using a younger sample. Nonetheless, depression can still develop at midlife and beyond, and many times, this is triggered by stressful life events, such as adverse social conditions.

### Subjective deprivation as a study limitation

A mechanism linking socioeconomic circumstances with depression involves subjective relative deprivation. Living in a deprived area can trigger comparison of the self to others, and this can in turn, lead to stress and poor mental health. A number of people living in deprived areas may experience negative emotions because they lack the necessary means to survive or are unable to achieve desired outcomes compared with those who are more affluent. Perceptions of lack can thus lead to poor health outcomes. Relative deprivation is composed of ‘affective and cognitive (ie, appraisal) responses to perceived unfair outcomes'.^61^ Thus, social comparisons and stress arising from deprivation can contribute to increased risk of depression. A recent study has indeed shown that subjective relative deprivation is linked to depressive symptoms.^61^ Living in a deprived area can give rise to subjective feelings of deprivation, which can subsequently lead to poor mental health. Although we did not have information on subjective feelings of deprivation, future studies should assess this.

### Future research

Future research should assess the risk of depression not only in regions, such as, the USA or UK where there is gender equality (more or less), but also in parts of the world where social roles and gendered norms for men and women have shown much less change over time. Interestingly, those countries with relatively high gender equality also show some of the highest rates of depression and other mental disorders in the world (though we do know a lot less about the state of mental health in low- and middle-income nations, so cross-cultural comparisons are difficult).^62^ Women have also been shown to be more affected by depression than men.^63^ More studies are needed to explore the influence of area deprivation on the mental health of men and women separately, and to do this in different contexts (eg, rural, urban) and countries around the world. Further, the reasons behind gender differences need to be better elucidated.

Finally, future studies should assess area deprivation and mental health at different points in time across the lifecourse and using a repeated measures' analysis, because both (deprivation and mental health) may change over an extended period of time.

### Placing our research in context^64^

Although other studies have shown that the places in which people live have a substantial impact on health,^14 15^ studies on the links between area deprivation and mental disorders from a gendered perspective are limited. A recent study^56^ of over 1000 African American and non-Hispanic white ("Black and White") adults living in the USA showed that men who experienced stressful life events in 1983–1986 were more likely to have depression in 2011, while this was not observed in women. This study, however, has limited generalisability, because it excluded other ethnicities. Also, the reliability and validity of the measure of stressful life events was not reported—the measure was based on a checklist of ‘major negative events’ that had occurred in the previous 3 years. Finally, exposure to stressful life events at the individual-level were investigated, rather than the effect of the place people live in.

A number of studies have assessed individual-level risk factors of depression, but substantially fewer have examined the influence of the environment on mental health. Nonetheless, studies of individual-level risk factors provide an important starting point in understanding relationships. Another prospective UK study of over 500 people^27^ showed that the socioeconomic status of men at midlife was associated with depression at midlife, while this was not observed in women. For women, their socioeconomic status at birth influenced their self-reported mental health at midlife. Also, men who had experienced downward social mobility or a reduction in their socioeconomic status from early adulthood to midlife were at a significantly increased risk of having poor mental health at midlife, but this was not observed in women.^27^ These results suggest that for women, the social class group they are in during early phases of life is important, while for men, social mobility over the life course, as well as the socioeconomic status group they are in during later life are crucial for their mental health. This study, however, was limited, because it was based on a small sample size, assessed only individual-level measures rather than area-level level effects, and failed to adjust for a number of important confounders, such as, demographic factors. Failure to properly adjust for potential confounders can lead to overestimation of the effect estimate. Finally, this study examined general mental health, rather than individual psychiatric disorders.

A recent US study showed that the types of stressors that influence men’s risk of depression are those related to work, finances, and legal matters.^25^ In this study, different types of stressors were linked to depression risk in women (and had less to do with economic/provider-role aspects). Again, this research only assessed individual-level data. Our study shows, for the first time that living in an area of above average deprivation increases the risk of depression in men, while less so in women.

### Interpretation

The genders seem to be differentially affected by the environment, and we believe it is important to highlight this for policy-makers, clinicians and public health authorities. Knowing that men living in areas of above average deprivation are more susceptible to depression can be used to inform treatment and prevention strategies—and knowing how to best tailor treatment efforts and targeted interventions is necessary at a time when there are insufficient health resources, such as now.
