# Supplementary material for: Association between area deprivation and major depressive disorder in British men and women: a cohort study
Source: BMJ Open. 2019 Nov 25;9(11):e027530. doi: 10.1136/bmjopen-2018-027530 (PMC6886936; doi:10.1136/bmjopen-2018-027530)
Supplement: Supplementary file 1 [file bmjopen-2018-027530supp001.pdf]

## Appendix 1

**Further information on A- and O-levels:**

A-levels are advanced level qualifications, which “are subject-based qualifications that can lead to university, further study, training, or work.” (UCAS)

According to IGCSE Centre, “O-Level is the abbreviation of Ordinary Level.  It is one of the two-part GCE (General Certificate of Education).  The other part of GCE is Advanced Level (A-Level), which students enter after completing O-Level.  O-Level is the final certification for secondary school, to be taken at fifth form or year 11 at approximately age 17 (or age group 14-16).  Students that have completed O-Level are considered to have completed formal education.” (IGCSE Centre)

## Appendix 2

***Distribution of Townsend index scores***

1. Above average deprivation

<u>Men</u>

Mean: 1.96

Standard deviation: 1.49

Range: 0.01 to 6.09

<u>Women</u>

Mean: 1.93

Standard deviation: 1.47

Range: 0.01 to 6.98

2. Below average deprivation

<u>Men</u>

Mean: -2.85

Standard deviation: 1.21

Range: -6.73 to -0.02

<u>Women</u>

Mean: -2.83

Standard deviation: 1.21

Range: -6.10 to -0.02

## Appendix 3

**Odds ratios for MDD according to individual- and area-level characteristics for men who completed the HLEQ questionnaire in the EPIC-Norfolk cohort**

| | Odds ratios and 95% CI | |
|---|---|---|
| **Characteristic*** | **Model C[1]** | **P-value for Model C** |
| **Individual-level variables** | | |
| **Socio-demographics** | | |
| **Age** | | |
| (per 10 years) | 0.47 (0.38, 0.59) | <0.0001 |
| **Education[‡]** | | |
| Low | 1.01 (0.69, 1.48) | 0.9420 |
| High | 1.00 | |
| **Marital status** | | |
| Single | 1.39 (0.71, 2.69) | <0.0001 |
| Married | 1.00 | |
| Other[*] | 3.51 (2.32, 5.29) | |
| **Social class[¥]** | | |
| Manual | 1.05 (0.75, 1.47) | 0.7747 |
| Non-manual | 1.00 | |
| **Employment[¥]** | | |
| No | 2.23 (1.45, 3.42) | 0.0002 |
| Yes | 1.00 | |
| **Health status** | | |
| **Lifetime GAD** | | |
| Yes | 12.65 (8.68, 18.43) | <0.0001 |
| No | 1.00 | |
| **Prevalent physical disease** | | |
| Yes[+] | 1.24 (0.88, 1.74) | 0.2176 |
| No | 1.00 | |
| **Disability level** | | |
| High[¶] | 2.01 (1.41, 2.86) | 0.0001 |
| Low | 1.00 | |
| **Area-level variable** | | |
| **Townsend index** | | |
| **Deprivation** | | |
| **Unemployment** | | |
| Yes (>0) | 1.77 (1.16, 2.71) | 0.0084 |
| No (<=0) | 1.00 | |
| **Non-car ownership** | | |
| Yes (>0) | 1.20 (0.70, 2.04) | 0.5067 |
| No (<=0) | 1.00 | |
| **Non-home ownership** | | |
| Yes (>0) | 0.81 (0.49, 1.34) | 0.4220 |
| No (<=0) | 1.00 | |

| | | |
|---|---|---|
| **Overcrowding** | | |
| Yes (>0) | 0.93 (0.60, 1.42) | 0.7272 |
| No (<=0) | 1.00 | |

1. Adjusted for age, sociodemographics, lifetime GAD, physical diseases and disability

‡ High education: O-level, A-level, degree; low education: refers to no education

* Other: divorced, separated, widowed

¥ Manual: skilled manual, semi-skilled, non-skilled; non-manual: professionals, managerial, skilled non-manual

+ Prevalent physical disease: respiratory disease (asthma, bronchitis), allergies (allergies, hay fever), stroke, heart attack, cancer, diabetes, thyroid conditions, arthritis

¶ Below the median PCS value of 50.6

*The brackets show the reference categories that were used for each categorical variable when it was entered in the models - below average deprivation [ref] vs. above average deprivation; education: high [ref] vs. low; marital status: married [ref], single, others; social class: non-manual [ref] vs. manual; employment: yes [ref] vs. no; lifetime GAD: no [ref] vs. yes; prevalent physical disease: no [ref] vs. yes; disability level: low [ref] vs. high. These reference categories were based on the literature.

**Appendix 4**

**Odds ratios for MDD according to individual- and area-level characteristics for men and women who completed the HLEQ questionnaire in the EPIC-Norfolk cohort**

| | Men (n=8,236) | | Women (n=10,335) | |
|---|---|---|---|---|
| | Odds ratios and 95% CI[1] | | Odds ratios and 95% CI[1] | |
| **Characteristic*** | | | | |
| **Individual-level variables** | | | | |
| **Socio-demographics** | | | | |
| **Age** | | | | |
| (per 10 years) | 0.47 (0.38, 0.59) | <0.0001 | 0.59 (0.51, 0.69) | <0.0001 |
| **Education**‡ | | | | |
| Low | 1.00 (0.69, 1.45) | 0.9977 | 1.30 (1.01, 1.67) | 0.0384 |
| High | 1.00 | | 1.00 | |
| **Marital status** | | | | |
| Single | 1.41 (0.72, 2.79) | 0.3181 | 0.91 (0.47, 1.75) | 0.7737 |
| Married | 1.00 | | 1.00 | |
| Other* | 3.58 (2.42, 5.28) | <0.0001 | 2.36 (1.85, 3.02) | <0.0001 |
| **Social class**¥ | | | | |
| Manual | 1.06 (0.76, 1.49) | 0.7340 | 0.97 (0.76, 1.24) | 0.8204 |
| Non-manual | 1.00 | | 1.00 | |
| **Employment**¥ | | | | |
| No | 2.24 (1.49, 3.37) | 0.0001 | 1.55 (1.18, 2.04) | 0.0019 |
| Yes | 1.00 | | 1.00 | |
| **Health status** | | | | |
| **Lifetime GAD** | | | | |
| Yes | 12.65 (8.71, 18.37) | <0.0001 | 7.37 (5.57, 9.75) | <0.0001 |
| No | 1.00 | | 1.00 | |
| **Prevalent physical disease** | | | | |
| Yes+ | 1.25 (0.90, 1.73) | 0.1842 | 1.25 (0.99, 1.59) | 0.0658 |
| No | 1.00 | | 1.00 | |
| **Disability level** | | | | |
| High¶ | 1.98 (1.38, 2.83) | 0.0002 | 1.41 (1.11, 1.80) | 0.0048 |
| Low | 1.00 | | 1.00 | |
| **Area-level variable** | | | | |
| **Townsend index** | | | | |
| **Deprivation** | | | | |
| Above average | 1.51 (1.03, 2.21) | 0.0358 | 1.24 (0.94, 1.64) | 0.1325 |

| | | |
|---|---|---|
| deprivation (>0) | | |
| Below average deprivation (<=0) | 1.00 | 1.00 |

1. Adjusted for age, sociodemographics, lifetime GAD, physical diseases and disability

[‡] High education: O-level, A-level, degree; low education: refers to no education

[*] Other: divorced, separated, widowed

[¥] Manual: skilled manual, semi-skilled, non-skilled; non-manual: professionals, managerial, skilled non-manual

[+] Prevalent physical disease: respiratory disease (asthma, bronchitis), allergies (allergies, hay fever), stroke, heart attack, cancer, diabetes, thyroid conditions, arthritis

[¶] Below the median PCS value of 50.6

*The brackets show the reference categories that were used for each categorical variable when it was entered in the models - below average deprivation [ref] vs. above average deprivation; education: high [ref] vs. low; marital status: married [ref], single, others; social class: non-manual [ref] vs. manual; employment: yes [ref] vs. no; lifetime GAD: no [ref] vs. yes; prevalent physical disease: no [ref] vs. yes; disability level: low [ref] vs. high. These reference categories were based on the literature.
